# Effects of Fucoidans on Activated Retinal Microglia

**DOI:** 10.3390/ijms25116018

**Published:** 2024-05-30

**Authors:** Philipp Dörschmann, Florentine Hunger, Hannah Schroth, Sibei Chen, Georg Kopplin, Johann Roider, Alexa Klettner

**Affiliations:** 1 Department of Ophthalmology, University Medical Center, University of Kiel, Arnold-Heller-Str. 3, Haus 25, 24105 Kiel, Germanyalexa.klettner@uksh.de (A.K.); 2 Alginor ASA, Haraldsgata 162, 5525 Haugesund, Norway; georg@alginor.no

**Keywords:** sulfated fucan, *Fucus vesiculosus*, *Laminaria hyperborea*, tumor necrosis factor alpha (TNFα), phagocytosis, gene expression, nitric oxide synthase 2 (*NOS2*), interleukin, toll-like receptor, lipopolysaccharide (LPS)

## Abstract

Sulfated marine polysaccharides, so-called fucoidans, have been shown to exhibit anti-inflammatory and immunomodulatory activities in retinal pigment epithelium (RPE). In this study, we tested the effects of different fucoidans (and of fucoidan-treated RPE cells) on retinal microglia to investigate whether its anti-inflammatory effect can be extrapolated to the innate immune cells of the retina. In addition, we tested whether fucoidan treatment influenced the anti-inflammatory effect of RPE cells on retinal microglia. Three fucoidans were tested (FVs from *Fucus vesiculosus*, Fuc1 and FucBB04 from *Laminaria hyperborea*) as well as the supernatant of primary porcine RPE treated with fucoidans for their effects on inflammatory activated (using lipopolysaccharide, LPS) microglia cell line SIM-A9 and primary porcine retinal microglia. Cell viability was detected with a tetrazolium assay (MTT), and morphology by Coomassie staining. Secretion of tumor necrosis factor alpha (TNFα), interleukin 1 beta (IL1β) and interleukin 8 (IL8) was detected with ELISA, gene expression (*NOS2* (Nitric oxide synthase 2), and *CXCL8* (IL8)) with qPCR. Phagocytosis was detected with a fluorescence assay. FucBB04 and FVs slightly reduced the viability of SIM-A9 and primary microglia, respectively. Treatment with RPE supernatants increased the viability of LPS-treated primary microglia. FVs and FucBB04 reduced the size of LPS-activated primary microglia, indicating an anti-inflammatory phenotype. RPE supernatant reduced the size of LPS-activated SIM-A9 cells. Proinflammatory cytokine secretion and gene expression in SIM-A9, as well as primary microglia, were not significantly affected by fucoidans, but RPE supernatants reduced the secretion of LPS-induced proinflammatory cytokine secretion in SIM-A9 and primary microglia. The phagocytosis ability of primary microglia was reduced by FucBB04. In conclusion, fucoidans exhibited only modest effects on inflammatorily activated microglia by maintaining their cell size under stimulation, while the anti-inflammatory effect of RPE cells on microglia irrespective of fucoidan treatment could be confirmed, stressing the role of RPE in regulating innate immunity in the retina.

## 1. Introduction

Inflammation is an important pathomechanism in degenerative retinal diseases such as age-related macular degeneration (AMD) [1]. AMD is the main cause of blindness in the elderly in the industrialized world and is expected to affect 288 million patients worldwide in 2040 [2]. It is considered a multifactorial disease with genetic, environmental, and lifestyle factors as contributing risk factors [1]. On a tissue level, the pathogenesis of AMD takes place at the photoreceptor/RPE/choroidal complex, with the RPE being considered to play a major part in AMD development [3]. Pathomechanisms include lipid accumulation, oxidative stress, proangiogenic signaling, and inflammation [1].

Due to the specific features of the retina, such as being hidden behind the blood–retina barrier and considered an immune privileged zone, the main players in retinal inflammation are the resident immune cell of the retina, the microglia and “border patrol” of the retina, and the retinal pigment epithelium (RPE), which contributes both to the immune privilege and the inflammatory responses [4,5]. As we and others have previously shown, proinflammatory activation of the RPE induces proinflammatory gene expression and (time-dependent) cytokine expression, such as long-term secretion of interleukin 6 (IL6) and interleukin 8 (IL8) and short-term secretion of IL1ß and TNFα [6,7,8,9,10,11,12]; loss of barrier function [13,14]; and loss of proteins important for RPE functions [8,15]. In addition, inflammation can reduce the viability of the RPE cells [10,16,17]. These data indicate that inflammation may contribute to AMD development by reducing the viability and functionality of RPE cells [18].

The main conductor of immune defense in the retina is the microglia. Its activation has been shown in several degenerative diseases of the retina, and its contribution to the pathology of AMD has been suggested in the literature [19,20,21]. Resting microglia, displaying an anti-inflammatory phenotype, are constantly surveying the retina. Upon stimulation, they change their phenotype, increasing in size, activating proinflammatory pathways, and secreting proinflammatory cytokines [22]. While a short-term activation to remove danger signals is vital to the retina, long-term or excessive activation may be harmful to the retinal neurons, contributing to neurodegeneration [23,24]. Interestingly, the activation of microglia can be ameliorated by activated RPE cells, reducing cytokine secretion and proinflammatory gene expression [25].

A potential new compound for the treatment of AMD is the sulfated polysaccharide of brown seaweed fucoidan, as antioxidative, antiangiogenic, and anti-inflammatory properties have been described [26]. However, the bioactivities of fucoidan are dependent on the species of origin, extracting methods, and chemical features, such as molecular weight [27,28]. Furthermore, the effect of fucoidans is cell-type specific [29]. We have previously shown in several studies that fucoidans of the seaweeds *Fucus vesiculosus* (FV) and *Laminaria hyperborea* show antioxidative, antiangiogenic, and anti-inflammatory effects on RPE cells [6,30,31,32,33]. The anti-inflammatory effect of fucoidans on RPE cells has recently been confirmed [34].

In this study, we investigated the effects of fucoidans from *Fucus vesiculosus* and *Laminaria hyperborea* on primary retina microglia and a microglia cell line, assessing viability, size, cytokine secretion, and gene expression in order to investigate whether the anti-inflammatory effects which fucoidans exert on RPE cells can be extrapolated to the retinal microglia. Furthermore, we also investigated the effect of fucoidan-treated RPE cells on the activation of microglia in order to assess whether the anti-inflammatory effect that the RPE exerts on microglia [25] is influenced (enhanced or diminished) when RPE cells are treated with fucoidan. Both aspects are of high interest if fucoidans are to be established as a potential therapy for the treatment of AMD.

## 2. Results

### 2.1. Iba-1 Staining

**Figure 1:**
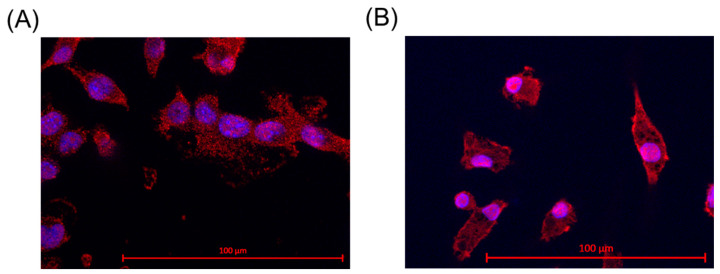
Iba-1 staining and proof for microglia. Ionized calcium binding adaptor molecule 1 (Iba-1) staining revealed Iba-1 expression (red marking) in the cells in SIM-A9 (**A**) and primary microglia (**B**). Objective = 63×, scale = 100 µm. Red = Iba-1, blue = nucleus.

The identity of microglia was assessed through ionized calcium binding adaptor molecule 1 (Iba-1) staining. This is a specific marker for microglia and macrophages [35]. SIM-A9 microglia and primary porcine microglia from the retinae were assessed. The results are shown in Figure 1. For primary microglia, positive cell nuclei and microglia were counted, and a nuclei/microglia ratio of 97.50% ± 2.59% was achieved. For SIM-A9, a ratio of 92.00% ± 9.80% was determined. Some microglia exhibited two nuclei, which is an indicator of cell division.

### 2.2. Cell Viability

The influence of fucoidans in inactive microglia and LPS-induced microglia regarding cell viability was tested. The fucoidans used are specified in Section 4.1. Several LPS concentrations, cell densities, and stimulation times were tested prior to determine the optimal conditions. Fucoidans in a concentration of 50 µg/mL were added 30 min prior to 1 µg/mL LPS treatment for 24 or 72 h.

**Figure 2:**
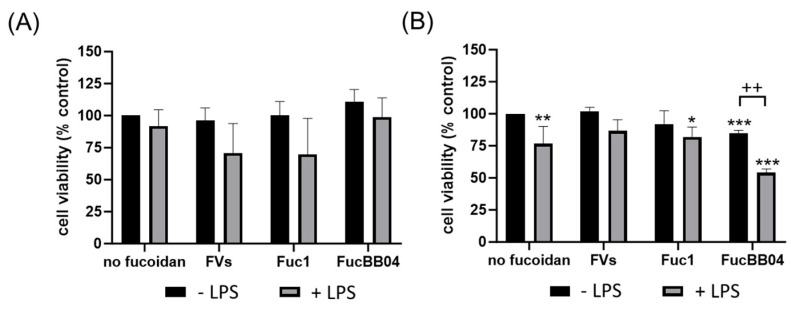
Effects of fucoidans and LPS on cell viability in SIM-A9. Mouse microglial cell line SIM-A9 was exposed to 50 µg/mL fucoidans FVs, Fuc1, and FucBB04 30 min prior to treatment with 1 µg/mL lipopolysaccharide (LPS) for 24 h (**A**) or 72 h (**B**). Tetrazolium bromide assay (MTT) was performed to measure cell viability. Cell survival was normalized to the untreated control, which was set to 100%. Mean and standard deviation of biological replicates are pictured. One-sample *t*-test was used to calculate significances against 100%. Student’s *t*-test was used to calculate significances compared to 1 µg/mL LPS. *n* = 4–8. *p*-values were calculated against 100% and LPS without fucoidans. * *p* < 0.05, ** *p* < 0.01, *** *p* < 0.001 compared to 100%. ++ *p* < 0.01 compared to 1 µg/mL LPS.

First, the mouse microglia cell line SIM-A9 was investigated (Figure 2). After 24 h of treatment (Figure 2A), the cell viability of SIM-A9 was not significantly reduced by any agent, but LPS treatment always exhibited a lower viability for each condition. Regarding 72 h of treatment (Figure 2B), single LPS (mean: 76.50% ± 13.72%, *p* = 0.002) or FucBB04 (mean: 85.00% ± 2.16%, *p* < 0.001) treatment decreased viability significantly. Combined treatment of Fuc1 + LPS reduced viability of SIM-A9 significantly (mean: 82.00% ± 7.75%, *p* = 0.020). Additionally, combined treatment of FucBB04 + LPS reduced viability significantly (mean: 54.00% ± 2.94%, *p* < 0.001), which was also significantly lower than single LPS treatment (*p* = 0.006).

**Figure 3:**
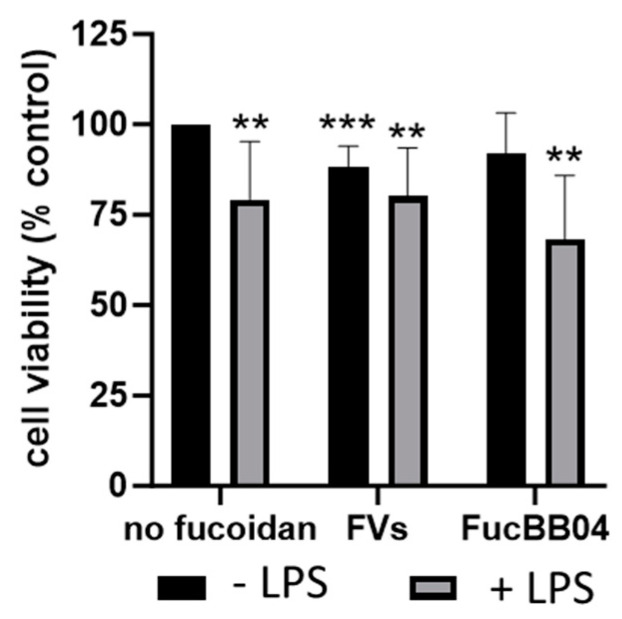
Effects of fucoidans and LPS on cell viability in primary microglia. Primary porcine microglia from retinae were exposed to 50 µg/mL fucoidans FVs and FucBB04 30 min prior to treatment with 1 µg/mL lipopolysaccharide (LPS) for 24 h. Tetrazolium bromide assay (MTT) was performed to measure cell viability. Cell survival was normalized to the untreated control, which was set to 100%. Mean and standard deviation of biological replicates are pictured. One-sample *t*-test was used to calculate significance against 100%. Student’s *t*-test was used to calculate significances compared to 1 µg/mL LPS. *n* = 8. *p*-values were calculated against 100% and LPS without fucoidans. ** *p* < 0.01, *** *p* < 0.001 compared to 100%. No significant differences compared to 1 µg/mL LPS were found.

Primary porcine microglia from pig retinae were stimulated with FVs and FucBB04, respectively, and/or LPS for 24 h (Figure 3). Besides single treatment with FucBB04, all conditions decreased cell viability significantly. LPS treatment reduced viability (mean: 79.25% ± 15.01%, *p* = 0.008), as did single treatment with FVs (mean: 88.13% ± 5.49%, *p* < 0.001). Combined treatment of FVs + LPS reduced viability (mean: 80.13% ± 12.49%, *p* = 0.004), as did FucBB04 + LPS, to the lowest value (mean: 68.25% ± 16.45%, *p* = 0.001), but these combined treatments were not significantly different from LPS alone.

**Figure 4:**
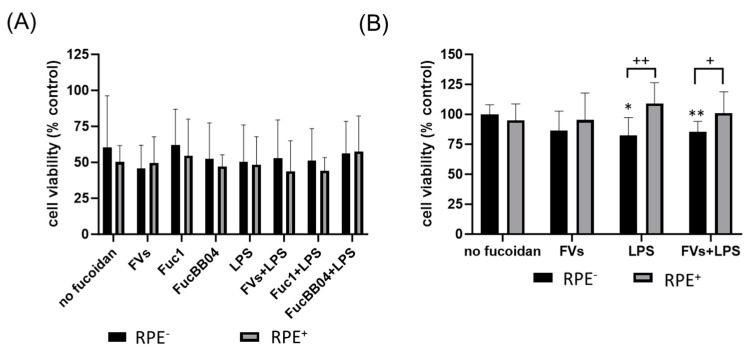
Effects of RPE supernatants on cell viability in SIM-A9 and primary microglia. Mouse microglia SIM-A9 (**A**) and primary porcine microglia from retinae (**B**) were exposed to 50 µg/mL fucoidans (FVs, Fuc1, and FucBB04 for SIM-A9 and FVs for primary microglia) 30 min prior to treatment with 1 µg/mL lipopolysaccharide (LPS) for 24 h (RPE^−^) or by instead using RPE supernatants (RPE^+^) stimulated (by the same agents) for three days. Tetrazolium bromide assay (MTT) was performed to measure cell viability. Cell survival was normalized to the untreated control, which was set to 100%. Mean and standard deviation of biological replicates are pictured. One-sample *t*-test was used to calculate significance against 100%. Student’s *t*-test was used to calculate significances compared to 1 µg/mL LPS. Paired Student’s *t*-test was used between RPE^−^ and RPE^+^ groups. *n* = 6. *p*-values were calculated against no fucoidan controls and LPS without fucoidans individually in the RPE^−^ and RPE^+^ group. Significances against the untreated control are not pictured. No significance to no fucoidan controls or LPS controls was determined. * *p* < 0.05, ** *p* < 0.01 compared to 100%; ^+^ *p* < 0.05, ^++^ *p* < 0.01 compared to RPE^−^. RPE^−^ = untreated RPE supernatant used for stimulation with agents, RPE^+^ = stimulated RPE supernatant was applied directly.

Finally, for both cell types, the influence of RPE supernatants was tested (Figure 4). A detailed explanation can be seen in Section 4.3. SIM-A9 (Figure 4A), and primary microglia (Figure 4B) were exposed to 50 µg/mL fucoidans (FVs^−^, Fuc1^−^ and FucBB04^−^ for SIM-A9 and FVs^−^ for primary microglia) 30 min prior to treatment with 1 µg/mL lipopolysaccharide (LPS) for 24 h (RPE^−^), or were stimulated with treated RPE supernatants (RPE^+^). Again, the MTT (tetrazolium bromide) assay was used for determining cell viability. Untreated cells were set as 100%. Significances were calculated against no fucoidan and LPS control with and without RPE treatment (RPE^−^, RPE^+^). Also, pairwise significances between matched RPE^−^ and RPE^+^ conditions were calculated. None of the tests performed in SIM-A9 showed any significant effects on viability. Regarding primary microglia, only the LPS (RPE^−^) (mean: 82.20% ± 13.66%, *p* = 0.030) and FVs + LPS (RPE^−^) (mean: 85.49% ± 7.94%, *p* = 0.010) conditions were significantly lower than the untreated control. No significant differences for no fucoidan controls or LPS controls were found, but pairwise comparison revealed that RPE^+^ supernatants led to significantly higher viability than using the direct stimulation of microglia with LPS (RPE^−^) (mean: 82.20% ± 13.66% to 109.23% ± 15.83%, *p* = 0.001) and FVs + LPS (RPE^−^) (mean: 85.49% ± 7.94% to 100.92% ± 16.48%, *p* = 0.016) conditions. Thus, RPE^+^ supernatants counteract LPS-induced reductions in viability.

### 2.3. Morphology

**Figure 5:**
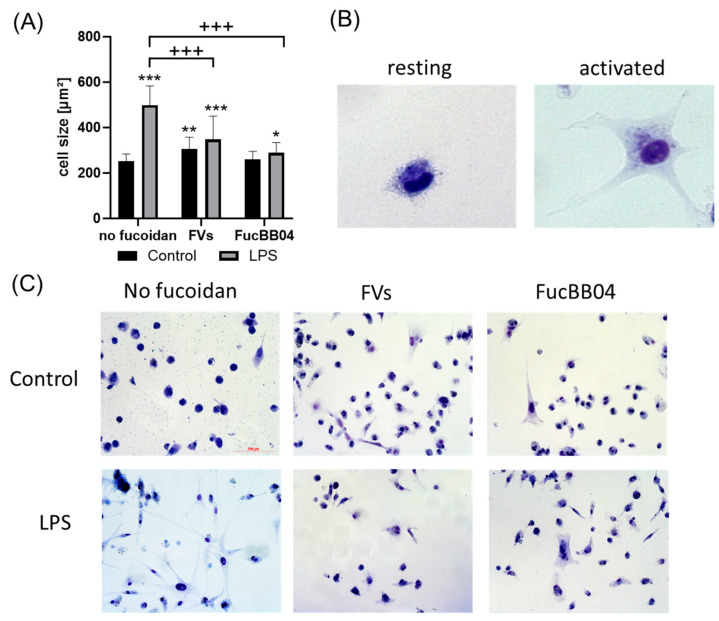
Effects of fucoidans and LPS on morphology in primary microglia. Primary porcine microglia from retinae were exposed to 50 µg/mL FVs and FucBB04 fucoidans 30 min prior to treatment with 1 µg/mL lipopolysaccharide (LPS) for 24 h. Cells were stained with Coomassie and images were taken by an inverse light microscope. Fiji was used to determine cell size in µm^2^ (**A**). Example pictures of resting and activated microglia are shown (**B**, objective = 20×, zoomed in). Mean and standard deviation of biological replicates are pictured. Example photos are given ((**C**), scale bar = 100 µm, objective = 20×). Kruskal–Wallis test followed by multiple comparison tests (Dunn’s test) were conducted to determine significances between groups. *n* = 30. *p*-values were calculated against untreated control or LPS control. * *p* < 0.05, ** *p* < 0.01, *** *p* < 0.001 compared to untreated control. ^+++^ *p* < 0.001 against 1 µg/mL LPS.

To measure the effects on the morphology and size of the microglia, Coomassie staining was applied [36]. Primary porcine microglia from retinae were stimulated with FVs, FucBB04, and/or LPS for 24 h, after which they were stained with Coomassie. Photos were taken using light microscopy and evaluated with Fiji (Facility for Imaging by Light Microscopy) by determining the cell area (Figure 5A). Example photos are shown (Figure 5B). Overall, an increased size of microglia correlates with proinflammatory activity [36]. Resting microglia are considered with an area of 100–499 µm^2^, and sizes over 499 µm^2^ are considered as activated [37]. The mean of resting microglia in this study was 221.41 ± 24.30 µm^2^. Besides FucBB04 single treatment, all conditions increased the size of the microglia significantly and thereby led towards an activated status, with LPS showing the strongest effect (mean: 522.40 ± 155.02 µm^2^, *p* < 0.001). FVs (mean: 337.89 ± 93.68 µm^2^, *p* = 0.002) and combined treatment of FVs + LPS (mean: 376.58 ± 93.93 µm^2^, *p* < 0.001), as well as FucBB04 + LPS (mean: 321.47 ± 111.69 µm^2^, *p* = 0.028), also exhibited increased cell size. Fucoidans showed counteracting effects by significantly reducing the LPS-induced size of the microglia (both *p* < 0.001).

**Figure 6:**
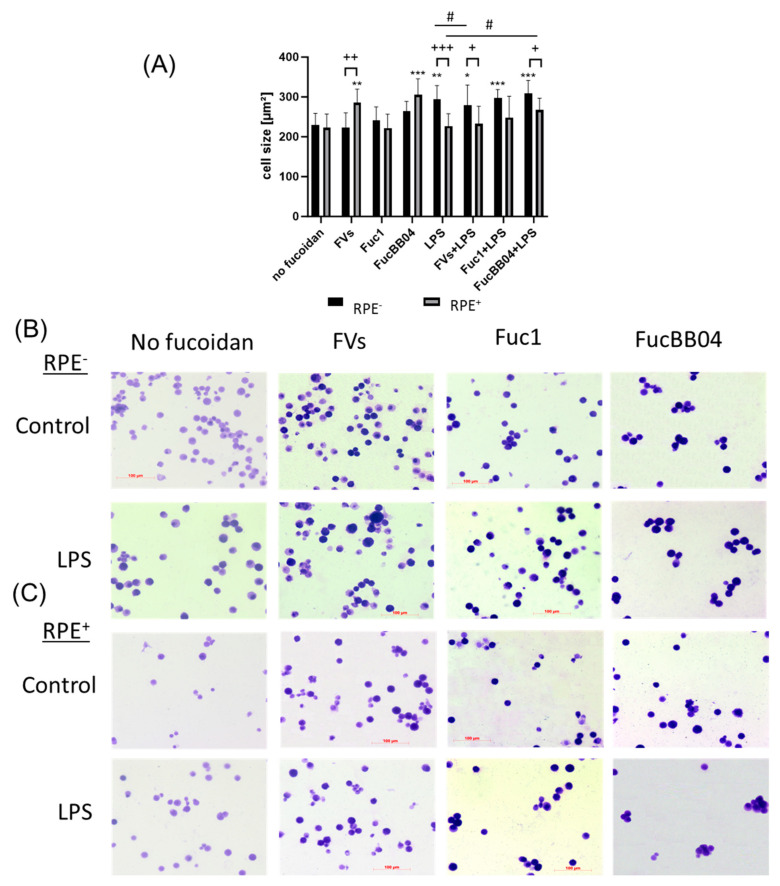
Effects of RPE supernatants on morphology in SIM-A9. Mouse microglia SIM-A9 were exposed to 50 µg/mL FVs, Fuc1, and FucBB04 30 min prior to treatment with 1 µg/mL lipopolysaccharide (LPS) for 24 h by using RPE supernatants (from the same plate) stimulated (by the same agents) for three days. (**A**) Cells were stained with Coomassie and images were taken by an inverse light microscope. Fiji was used to determine cell size in µm^2^. Mean and standard deviation of biological replicates are pictured. Kruskal–Wallis test followed by multiple comparison tests (Dunn’s test) were conducted to determine significances between groups. *n* = 9. *p*-values were calculated against no fucoidan controls and LPS controls individually in the RPE^−^ and RPE^+^ group. * *p* < 0.05, ** *p* < 0.01, *** *p* < 0.001 compared to no fucoidan controls. ^#^ *p* < 0.05 compared to LPS. ^+^ *p* < 0.05, ^++^ *p* < 0.01, ^+++^ *p* < 0.001 compared to RPE^−^. RPE^−^ = untreated RPE supernatant used for stimulation with agents; RPE^+^ = stimulated RPE supernatant was applied directly. Example photos are given ((**B**) = directly stimulated cells (RPE^−^); (**C**) = cells treated with RPE supernatants (RPE^+^); scale bar = 100 µm, objective = 20×).

In addition, the influence of RPE supernatants was tested for SIM-A9 (Figure 6). The cells were exposed to 50 µg/mL FVs, Fuc1, and FucBB04 30 min prior to treatment with 1 µg/mL lipopolysaccharide (LPS) for 24 h (RPE^−^) or by instead using RPE supernatants (RPE^+^) stimulated (by the same agents) for three days. Coomassie staining was used to stain the cells and Fiji to measure cell size (Figure 6A). Example photos are given (Figure 6B,C). Untreated resting SIM-A9 showed a size of 229.75 ± 29.06 µm^2^. Again, the size of the cells increased with proinflammatory activation, with LPS (RPE^−^) treatment increasing the cell size (mean: 294.07 ± 34.22 µm^2^, *p* = 0.001). The morphological changes regarding inflammatory activation of SIM-A9 were not as strong as in primary microglia. The LPS (RPE^+^) supernatant lost this significant effect (compared to no fucoidan control), and the cell size was lowered significantly (mean: 226.78 ± 31.22 µm^2^, *p* < 0.001). The same pattern was detected for FVs + LPS (RPE^+^) (mean: 278.80 ± 51.55 µm^2^ to 232.98 ± 44.03 µm^2^, *p* = 0.024) and FucBB04 + LPS (RPE^+^) (mean: 309.62 ± 32.07 µm^2^ to 267.25 ± 29.85 µm^2^, *p* = 0.016), which were also significantly reduced by RPE treatment. Interestingly, FV (RPE^+^) cell size after treatment was significantly increased due to RPE treatment compared to direct FV (RPE^−^) treatment (mean: 223.01 ± 36.99 µm^2^ to 285.05 ± 34.67 µm^2^, *p* = 0.008). Overall, our data indicate a general anti-inflammatory effect of RPE in microglia. Also, the FVs + LPS (RPE^−^)-treated cell size was significantly lower than the LPS (RPE^−^) control (from mean: 294.07 µm^2^ ± 34.22 µm^2^ to 278.80 µm^2^ ± 51.55 µm^2^, *p* = 0.0179), and FucBB04 + LPS (RPE^+^) led to a significantly higher cell size than the LPS (RPE^+^) control (from mean: 226.78 ± 31.22 µm^2^ to 248.63 µm^2^ ± 29.85 µm^2^).

### 2.4. Cytokine Secretion

The influence of fucoidans on unstimulated microglia and LPS-stimulated microglia on cytokine secretion was tested. The tested fucoidans are specified in Section 4.1. Several LPS concentrations, cell densities, and stimulation times were tested prior to determining optimal conditions. Fucoidans in a concentration of 50 µg/mL were added 30 min prior to 1 µg/mL LPS treatment for 24 or 72 h. ELISA data were normalized with cell viability data from Section 2.2.

**Figure 7:**
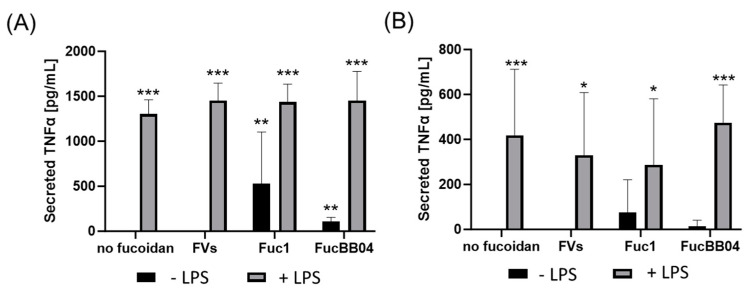
Effects of fucoidans and LPS on TNFα secretion in SIM-A9. Mouse microglial cell line SIM-A9 was exposed to 50 µg/mL of fucoidans FVs, Fuc1, and FucBB04 30 min prior to treatment with 1 µg/mL lipopolysaccharide (LPS) for 24 h (**A**) or 72 h (**B**). ELISA was performed to measure secreted tumor necrosis factor alpha (TNFα). Secreted protein was normalized to cell viability. Mean and standard deviation of biological replicates are pictured. ANOVA (analysis of variance) with post hoc multiple comparison tests (Dunnett’s test) was used to calculate significances between groups. *n* = 7–21. *p*-values were calculated against untreated control and LPS. * *p* < 0.05, ** *p* < 0.01, *** *p* < 0.001 compared to control. No significance against 1 µg/mL LPS was determined.

First, the mouse microglia cell line SIM-A9 was investigated (Figure 7). Supernatants were collected for 24 h and applied in ELISA to measure secreted tumor necrosis factor alpha (TNFα). After 24 h of treatment (Figure 7A), LPS led to a significant secretion of TNFα (mean: 1301.93 ± 158.72 pg/mL, *p* < 0.001). This was not reduced by fucoidans. Furthermore, single treatment with Fuc1 (mean: 435.82 ± 482.52 pg/mL, *p* = 0.005) or FucBB04 (mean: 106.21 ± 48.44 pg/mL, *p* = 0.001) induced TNFα secretion after 24 h. Also, combined treatments of FVs + LPS (mean: 1453.33 ± 193.11 pg/mL, *p* < 0.001), Fuc1 + LPS (mean: 1435.44 ± 198.31 pg/mL, *p* < 0.001), and FucBB04 + LPS (mean: 1452.67± 324.04 pg/mL, *p* < 0.001) induced TNFα secretion.

After 72 h of SIM-A9 stimulation (Figure 7B), LPS (mean: 419.11 ± 293.47 pg/mL, *p* < 0.001), FVs + LPS (mean: 329.25 ± 278.66 pg/mL, *p* = 0.012), Fuc1 + LPS (mean: 286.94 ± 293.76 pg/mL, *p* = 0.028), and FucBB04 + LPS (mean: 474.06 ± 168.18 pg/mL, *p* < 0.001) significantly increased TNFα secretion, but all to a lower degree compared to 24 h. The effect of fucoidans was transient, as after 72 h of stimulation, fucoidans no longer showed a significant effect on TNFα secretion. No significant changes in TNFα secretion were achieved with fucoidan in any combination with LPS compared to LPS alone for 24 and 72 h. Notably, numerically, FVs and Fuc1 combined with LPS showed lower values and FucBB04 higher values then single treatment with LPS.

**Figure 8:**
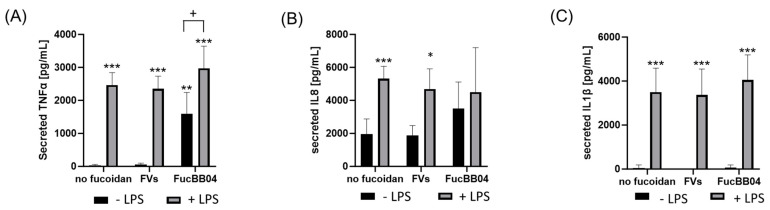
Effects of fucoidans and LPS on TNFα, IL8, and IL1β secretion in primary microglia. Primary porcine microglia from retinae were exposed to 50 µg/mL fucoidans FVs and FucBB04 30 min prior to treatment with 1 µg/mL lipopolysaccharide (LPS) for 24 h. ELISA was performed to measure secreted tumor necrosis factor alpha (TNFα, (**A**)), interleukin 8 (IL8, (**B**)), and interleukin 1 beta (IL1β, (**C**)). Secreted protein was normalized to cell viability. Mean and standard deviation of biological replicates are pictured. ANOVA (analysis of variance) with post hoc multiple comparison tests (Dunnett’s test) was used to calculate significances between groups. *n* = 8. *p*-values were calculated against untreated control and LPS. * *p* < 0.05, ** *p* < 0.01, *** *p* < 0.001 compared to control. ^+^ *p* < 0.05 compared to 1 µg/mL LPS.

In addition, primary porcine microglia from retinae were tested (Figure 8). They were treated with LPS and FVs or FucBB04, and the secretion of TNFα, IL8 and IL1β was assessed. Regarding TNFα (Figure 8A), LPS induced TNFα secretion (mean: 2466.88 ± 343.44 pg/mL, *p* < 0.001). Single treatment with FucBB04 activated microglia (mean: 1599.90 ± 584.60 pg/mL, *p* = 0.006). Combined stimulation of FVs + LPS (mean: 2353.52 ± 348.45 pg/mL, *p* < 0.001) and FucBB04 + LPS (mean: 2974.62 ± 612.17 pg/mL, *p* < 0.001) increased TNFα secretion as well. Combined treatment of FucBB04 + LPS was significantly higher than FucBB04 itself (*p* = 0.021). 

Concerning IL8 (Figure 8B), there was a basal IL8 secretion with 1952.56 ± 842.29 pg/mL and LPS-activated cytokine secretion compared to control (mean: 5324.80 ± 680.81 pg/mL, *p* < 0.001). LPS + FVs also showed significant higher IL8 secretion compared to the control (mean: 4696.34 ± 1116.45 pg/mL, *p* < 0.0291), but it was numerically lower than LPS alone. Also, FucBB04 + LPS showed no significant effect compared to LPS alone.

Concerning IL1β secretion (Figure 8C) LPS treatment showed significant induction of cytokines (mean: 3496.04 ± 1019.23 pg/mL, *p* < 0.001). Combined treatments of FVs + LPS (mean: 3372.89 ± 1096.69 pg/mL, *p* < 0.001) and FucBB04 + LPS (mean: 4050.45 ± 1068.14 pg/mL, *p* < 0.001) activated IL1β as well. Combination of fucoidan with LPS did not display significant differences compared to LPS alone.

**Figure 9:**
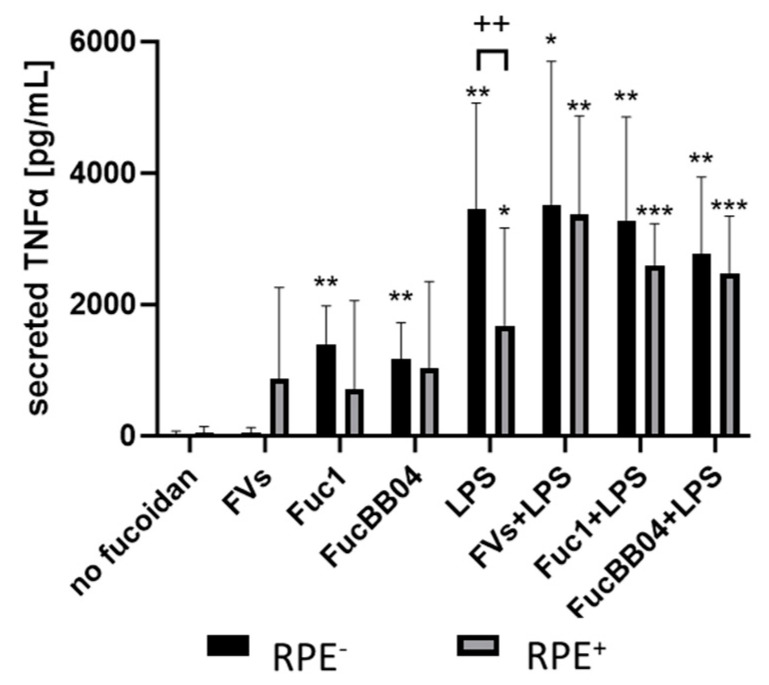
Effects of RPE supernatants on TNFα secretion in SIM-A9. Mouse microglia SIM-A9 were exposed to 50 µg/mL FVs, Fuc1, and FucBB04 30 min prior to treatment with 1 µg/mL lipopolysaccharide (LPS) for 24 h by using retinal pigment epithelium (RPE) supernatant (collected for three days) or by using instead RPE supernatants (from same plate) stimulated (by the same agents) for three days. ELISA was performed to measure secreted tumor necrosis factor alpha (TNFα). Secreted protein was normalized to cell viability. Mean and standard deviation of biological replicates are pictured. ANOVA (analysis of variance) with post hoc multiple comparison tests (Dunnett’s test) was used to calculate significances between groups. Paired Student’s *t*-test was used between RPE^−^ and RPE^+^ groups. *n* = 6. *p*-values were calculated against untreated control and LPS individually in the RPE^−^ and RPE^+^ groups. * *p* < 0.05, ** *p* < 0.01, *** *p* < 0.001 compared to control. No significance against 1 µg/mL LPS controls was determined. ^++^ *p* < 0.01 compared to RPE^−^. RPE^−^ = untreated RPE supernatant used for stimulation with agents; RPE^+^ = stimulated RPE supernatant was applied directly.

**Figure 10:**
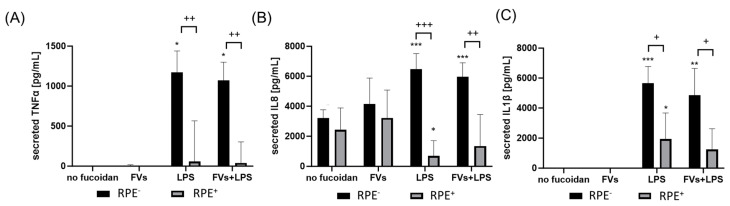
Effects of RPE supernatants on TNFα, IL8 and IL1β secretion in primary microglia. Primary porcine microglia from retinae were exposed to 50 µg/mL fucoidans FVs 30 min prior to treatment with 1 µg/mL lipopolysaccharide (LPS) for 24 h by using retinal pigment epithelium (RPE) supernatant (collected for three days) or by using instead RPE supernatants (from same plate) stimulated (by the same agents) for three days. ELISA was performed to measure secreted tumor necrosis factor alpha (TNFα, (**A**)), interleukin 8 (IL8, (**B**)), and interleukin 1 beta (IL1β, (**C**)). Secreted protein was normalized to cell viability, and RPE cytokine content was subtracted. Mean and standard deviation of biological replicates are pictured (for TNFα mean and standard deviation). ANOVA (analysis of variance) with post hoc multiple comparison tests (Dunnett’s test) was used to calculate significances between groups. Paired Student’s *t*-test was used between the RPE^−^ and RPE^+^ groups. *n* = 6. *p*-values were calculated against untreated control and LPS individually in the RPE^−^ and RPE^+^ groups. * *p* < 0.05, ** *p* < 0.01, *** *p* < 0.001 compared to control. No significance against 1 µg/mL LPS controls was determined. ^+^ *p* < 0.05, ^++^ *p* < 0.01, ^+++^ *p* < 0.001 compared to RPE^−^. RPE^−^ = untreated RPE supernatant used for stimulation with agents, RPE^+^ = stimulated RPE supernatant was applied directly.

Finally, for both cell types, the influence of RPE supernatants was tested. SIM-A9 (Figure 9) and primary microglia (Figure 10) were exposed to 50 µg/mL fucoidans (FVs, Fuc1, and FucBB04 for SIM-A9 and FVs for primary microglia) 30 min prior to treatment with 1 µg/mL LPS for 24 h (RPE^−^) or by instead using RPE supernatants (RPE^+^) stimulated (by the same agents) for three days.

Regarding SIM-A9, LPS (RPE^−^)-induced TNFα secretion (mean: 3449.14 ± 1478.41 pg/mL, *p* = 0.004) was significantly reduced by the RPE^+^-treated supernatant with LPS (RPE^+^) (1683.20 ± 1356.02 pg/mL, *p* = 0.045). Also, with the exception of single treatment with FVs, all conditions were numerically reduced if RPE-conditioned stimuli were used. No significant effects of combined treatments with LPS or single LPS treatment were detected (both RPE^−^, RPE^+^).

Regarding primary microglia, LPS (RPE^−^) (mean: 1202.57 ± 194.41 pg/mL, *p* = 0.031) and FVs + LPS (RPE^−^) (mean: 1138.64 ± 116.06 pg/mL, *p* = 0.031) induced TNFα secretion significantly. This was significantly diminished by using RPE^+^ treated supernatants for LPS (RPE^+^) (mean: 241.07 ± 323.70 pg/mL, *p* = 0.003) and FVs + LPS (RPE^+^) (mean: 176.96 ± 311.14 pg/mL, *p* = 0.002).

Regarding IL8, a basal secretion was detected (mean: 3217.98 ± 496.33 pg/mL), which was slightly numerically reduced by using RPE^+^ supernatant (mean: 2434.63 ± pg/mL). Compared to the control, LPS (RPE^−^) (mean: 6469.07 ± 946.03 pg/mL, *p* = 0.001) and FVs + LPS (RPE^−^) (mean: 5974.31 ± 842.95 pg/mL, *p* < 0.001) enhanced IL8 secretion, whereas the LPS (RPE^+^)-treated RPE supernatant reduced it remarkably compared to the control (mean: 649.62 ± 962.20 pg/mL, *p* = 0.004) and compared to LPS (RPE^−^) (*p* < 0.001). Also, by using the RPE^+^ supernatant, FVs + LPS (RPE^+^) showed significantly lower IL8 secretion (mean: 1219.79 ± 2005.43 pg/mL, *p* = 0.008) than with RPE^−^ counterpart.

Concerning IL1β secretion, LPS (RPE^−^) induced cytokine secretion (mean: 5649.54 ± 1019.79 pg/mL, *p* < 0.001), as did FVs + LPS (RPE^−^) (mean: 4863.79 ± 1623.79 pg/mL, *p* = 0.001) and LPS (RPE^+^) (mean: 1934.87 ± 1581.18 pg/mL, *p* = 0.041). Using RPE^+^ supernatants reduced this secretion for LPS (RPE^+^) (from mean: 5649.54 ± 1019.79 pg/mL to mean: 1934.87 ± 1581.18 pg/mL, *p* = 0.013) and FVs + LPS (RPE^+^) (from mean: 4863.79 ± 1623.79 pg/mL to mean: 1245.10 ± 1259.23 pg/mL, *p* = 0.027).

### 2.5. Gene Expression

Inflammation-relevant gene expression in microglial cells was investigated by qPCR using different marker genes depending on the cell type. Several LPS concentrations, cell densities, genes, and stimulation times were tested prior to the actual fucoidan testing to determine the optimal conditions.

Regarding SIM-A9, *NOS2* expression (gene for Nitric oxide synthase 2) was assessed in non-activated and LPS-induced cell samples (Table 1). The expression of *GAPDH* was used for normalization. Fucoidans, at a concentration of 50 µg/mL, were added 30 min prior to 1 µg/mL LPS treatment for 24 or 72 h. After 24 h of stimulation, LPS increased *NOS2* gene expression significantly compared to control (Rq: 7.422, *p* = 0.035). Combined stimulation of FucBB04 + LPS showed even stronger effects than LPS alone (Rq: 9.229, *p* = 0.018). After 72 h of stimulation, again, *NOS2* expression of LPS was significantly higher than the control (Rq: 6.015, *p* = 0.006). Combined treatment of Fuc1 + LPS showed a significant *NOS2* increase (Rq: 8.056, *p* = 0.002). The case was the same for FVs + LPS (Rq: 4.013, *p* = 0.017). Compared to LPS, no significances were found.

In primary porcine microglia (Table 2) LPS showed significantly higher gene expression of *CXCL8* (interleukin 8) (Rq: 109.550, *p* = 0.005) than the control, as well as FVs + LPS (Rq: 137.650, *p* = 0.004) and FucBB04 + LPS (Rq: 659.110, *p* = 0.001). *NOS2* gene expression was significantly higher with LPS (Rq: 20.460, *p* = 0.009) and FVs + LPS (Rq: 28.850, *p* = 0.005). Again, no significances against LPS were determined.

Finally, the influence of RPE supernatants was tested (Table 3). SIM-A9 was exposed to 50 µg/mL FVs or FucBB04 30 min prior to treatment with 1 µg/mL lipopolysaccharide (LPS) for 24 h (RPE^−^), or by applying RPE supernatants (RPE^+^) stimulated (by the same agents) for three days instead. *NOS2* gene expression was detected and normalized with *GAPDH* expression. Control or LPS was set to Rq = 1.000. *p*-values were calculated against the control (RPE^−^), control (RPE^+^), LPS (RPE^−^), and LPS (RPE^+^) groups. Compared to the untreated control (RPE^−^) several conditions showed increased *NOS2* expression with FVs (RPE^−^) (Rq: 2.371, *p* = 0.047), FucBB04 (RPE^−^) (Rq: 5.011, *p* = 0.048), LPS (RPE^−^) (Rq: 41.193, *p* < 0.001), FVs + LPS (RPE^−^) (Rq: 30.788, *p* < 0.001), FucBB04 + LPS (RPE^−^) (Rq: 22.226, *p* = 0.002), LPS (RPE^+^) (Rq: 17.843, *p* = 0.002), FVs + LPS (RPE^+^) (Rq: 43.195, *p* = 0.001), and FucBB04 + LPS (RPE^+^) (Rq: 23.564, *p* = 0.001). All these conditions showed the same significant effects if calculated against the control (RPE^+^) (refer Table 3). FVs (RPE^+^) and FucBB04 (RPE^+^) lost their significant NOS increases when using RPE supernatants. No significances against LPS (RPE^−^) or LPS (RPE^+^) were calculated.

### 2.6. Phagocytosis

**Figure 11:**
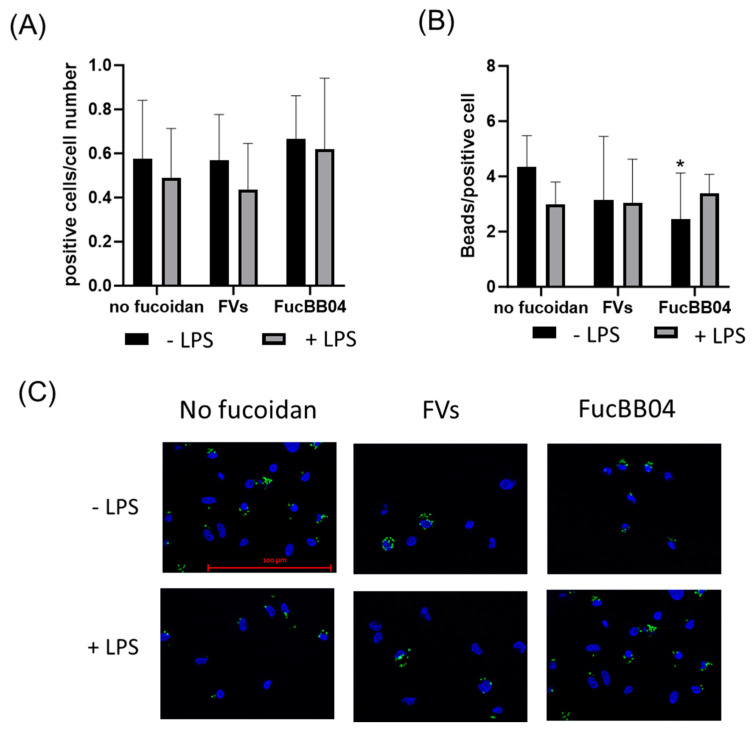
Effects of fucoidans and LPS on phagocytosis activity in primary microglia. Primary porcine microglia from retinae were exposed to 50 µg/mL fucoidans FVs or FucBB04 30 min prior to treatment with 1 µg/mL lipopolysaccharide (LPS) for 24 h. Fluorescence beads were applied for two hours. Cell nuclei and beads were determined by fluorescence imaging and Fiji evaluation. The number of cells with at least one internalized bead was set in relation to the total cell number in the photo (**A**), and the total bead number in the photo was divided by the number of all positive cells in the photo (**B**). Mean and standard deviation of biological replicates are pictured. Example photos are given ((**C**), scale bar = 100 µm, 63×). Kruskal–Wallis test followed by multiple comparison tests (Dunn’s test) were conducted to determine significances between groups. *n* = 30. *p*-values were calculated against untreated control or LPS control. * *p* < 0.05 compared to untreated control. No significance against 1 µg/mL LPS was determined.

Primary porcine microglia from retinae were treated with FVs, FucBB04, and/or LPS for 24 h. Then, fluorescence-labeled latex beads were applied. The number of cells with at least one internalized bead was set in relation to the total cell number in the photo (Figure 11A), and the total bead number in the photo was divided by the number of all positive cells in the photo (Figure 11B). Nearly half of all microglia cells phagocyted at least one bead and were positive (positive cells range from mean: 0.44 ± 0.20 [arb. unit] (FVs + LPS) to mean: 0.67 ± 0.19 [arb. unit] (E127)) with no significant differences, but nominally, all LPS-treated conditions were lowered. The number of beads that were phagocyted by all microglia is a measure of phagocytic ability. Compared to the untreated control and LPS control, no significant differences were determined, with the exception of FucBB04 treatment, with a lowered bead number per cell (mean: 2.45 ± 1.57 [arb. unit], *p* = 0.023) compared to the untreated control (mean: 4.33 ± 1.98 [arb. unit]).

## 3. Discussion

For multifactorial diseases like age-related macular degeneration [1], new therapeutics that target several of the pathomechanisms would open a new avenue for AMD treatment, focusing on the early stages and limiting progression [27]. We have previously shown that fucoidans from *Fucus vesiculosus* and *Laminaria hyperborea* exhibit antiangiogenic, antioxidative, and anti-inflammatory properties on RPE cells [6,30,31,32,33]. In this study, we focused on the anti-inflammatory effects of these fucoidans on microglia cells using both a microglial cell line and primary porcine RPE cells. We investigated several aspects of microglial activation (induced by LPS), investigating cell size as an indicator of activation, cytokine secretion, gene expression, and phagocytosis, which were all previously established for retinal microglia [25,36,37].

This is of particular interest, as the effect of fucoidans is strongly dependent not only on the specific fucoidan, but also on the cell type and model system used (e.g., [29,33]). For instance, in previous studies focusing on the effects of different fucoidans on macrophages, both pro- and anti-inflammatory effects were found [38,39,40,41,42]. The literature on the effect of fucoidans on microglia indicates a general anti-inflammatory effect [43,44,45,46]; however, these studies were focusing on brain microglia. It is important to keep in mind that the bioactivity of fucoidan is dependent on its origin and chemical properties [47]. In addition, fucoidans can exert different effects in different cell types, including macrophages and microglia [29,38]; therefore, each fucoidan needs to be tested in the relevant cell types. The effect found in one cell type cannot be transferred to another cell type without experimental evidence. To our knowledge, this is the first study investigating the effect of fucoidan on retinal microglia, for which we used primary porcine retinal microglia [25,36,37]. Primary microglia were prepared from the retinae of individual pigs that were kept according to farm husbandry, not under sterile laboratory conditions. Therefore, the primary microglia used for experimentation are genetically heterogeneous (in contrast to cell lines or inbred mouse strains) and from different “life styles” (housing conditions). This may have led to a higher standard deviation than what the reader is used to when assessing data obtained from genetically homogeneous cell lines or inbred mouse strains kept under laboratory conditions. However, this kind of data may reflect the “real-world” situation more closely, as patients are likewise genetically diverse and live according to different lifestyles.

In addition, we utilized the murine brain-derived microglia cell line SIM-A9 [48], which has been used in several studies of LPS-induced inflammation [49,50,51]. While the SIM-A9 cell line is an important model for microglia, it is a cell line (as compared to primary microglia) derived from the brain (not from the retina), and is of a different species than the RPE cells used in this study (murine vs. porcine). These factors need to be considered when interpreting the results obtained in this study.

While we investigated several aspects of inflammatory microglia activation, a general anti-inflammatory effect of fucoidans on retinal microglia cannot be claimed according to our data. Especially concerning cytokine release, no reduction caused by fucoidan could be found. This strongly indicates that microglia activation is not inhibited by the fucoidans tested. However, our data indicate a modest and specific anti-inflammatory effect of some fucoidans on primary retinal microglia, which justifies further investigation. Specifically, fucoidans significantly inhibited the increase in cell size induced by LPS, indicating that fucoidans help to maintain an anti-inflammatory phenotype of the cells [36]. This finding is in accordance with previous studies, which showed that 62.5 µg/mL fucoidan from *Laminaria japonica* attenuated the morphological changed induced by LPS in microglia derived from rat brains [45]. Similar results on the influence on morphology were obtained by the same group in in vivo [43]. Interestingly, these authors also found a reduction in iNOS mRNA expression, but at higher concentrations (125 µg/mL fucoidan) than those we used in our study. This might indicate that the influence on morphology is less dependent on the species and concentration than the specific influence on the expression of certain genes. On the other hand, in a study by Park and al., a reduction in iNOS expression was seen in a BV2 murine microglia cell line already at 25 µg/mL with fucoidan from *Fucus vesiculosus*. However, the data shown were only qualitative according to normal PCR, and no quantification or statistical analysis was conducted [44]. The same study showed a decrease in TNFα secretion, but at a higher concentration compared to our study (100 µg/mL). Further studies have to be conducted to test other fucoidans and other parameters to confirm whether this effect can be considered general for retina microglia or whether it is a specific effect of these tested fucoidans under these test parameters. In addition, additional aspects of microglia activation would be of interest, such as cytoplasmic makers, further pro-inflammatory gene expression, or additional interleukin or chemokine secretion. For example, it would be of interest whether the effect would be similar if a Toll-like-receptor (TLR3) antagonist were used, as we have previously shown that several fucoidans can reduce inflammatory activation of RPE cells after TLR-3 activation [30,52]. Also, different timelines of activation and concentrations of fucoidan would be of interest. Finally, all aspects that are covered in vitro should be assessed in vivo, such as AMD mouse models [53,54].

In addition, additional aspects of microglia activation would be of interest, such as cytoplasmic makers, further pro-inflammatory gene expression, or additional interleukin or chemokine secretion.

The induction of cytokines such as TNFα and pro-inflammatory genes such as *NOS2* under fucoidan treatment also needs to be investigated. It is of interest that these effects are reproductive and significant, but the grade of induction is far below that which is seen with a pro-inflammatory activator such as LPS, and is lost when LPS is applied. The effects of this modest activation of microglia on the retina need to be further investigated.

As our main target for AMD treatment is the RPE, in addition to investigating the effects of fucoidans alone, we also assessed the effect of (fucoidan-treated) RPE cells. We have previously shown that pro-inflammatorily activated RPE cells reduce the pro-inflammatory activation of microglia, reducing its cytokine secretion and pro-inflammatory gene expression [25]. This was confirmed in this study and could be extended to fucoidan-treated RPE cells. Our data clearly indicate an attenuating effect of RPE cells on microglia, stressing the importance of RPE in the downregulation of retinal responses to danger signals.

Taken together, our results show that the anti-inflammatory effects of the tested fucoidans on RPE cells cannot be extrapolated on microglia, but that the anti-inflammatory effect RPE cells display on microglia persist under fucoidan treatment.

## 4. Material and Methods

### 4.1. Fucoidans

To test the biological activities of fucoidans in microglia, three fucoidan samples were used (Table 4). FVs was purchased from Sigma-Aldrich (St. Louis, MO, USA), and Fuc1 and FucBB04 were provided by Georg Kopplin from Alginor ASA (Haugesund, Norway). Fucoidan properties are depictured in Table 4. All fucoidans can be considered as high-molecular weight fucoidans [55] and as pure, with at least 86% fucose. The exact chemical and structural properties of the fucoidans are described elsewhere [31,32,56]. A manuscript concerning the chemical characteristics of FucBB04 is currently in the preparation stages.

### 4.2. Cell Culture

Primary RPE cells were prepared from porcine eyes as previously described [57]. Eyes were obtained from local slaughterhouses as byproducts of the food industry. All experiments were conducted in accordance with the animal welfare officer of University of Kiel and are considered as an active contribution to reducing animal experiments according to the 3R principle [58]. In brief, the eyes were disinfected and cleaned, and the cornea, iris, lens, and vitreous body were removed. RPE cells were detached by incubation with trypsin (Pan-Biotech, Aidenbach, Germany) followed by trypsin/EDTA (Pan-Biotech), then were washed with media and seeded into 12-well plates (Sarstedt, Nümbrecht, Germany). Media contained Gibco DMEM (Thermo Fisher Scientific, Waltham, CA, USA), 10% fetal bovine serum (Thermo Fisher Scientific), 1% penicillin/streptomycin (Sigma-Aldrich, St. Louis, MO, USA), 2.5% HEPES (Pan-Biotech), and 1% non-essential amino acids (Pan-Biotech). RPE cells were cultured for at least two weeks before stimulation.

The mouse microglial cell line SIM-A9 was purchased from American Type Culture Collection (ATCC, Manassas, VA, USA). Culturing was performed as recommended by ATCC. The media consisted of DMEM/F12 (Pan-Biotech) with heat-inactivated 10% fetal bovine serum and heat-inactivated 5% horse serum (Sigma-Aldrich). For sub-culturing, cells were incubated with Ca^2+^/Mg^2+^-free Dulbecco’s phosphate-buffered saline (D-PBS, Pan-Biotech), 1 mM EDTA (Sigma-Aldrich), 1 mM EGTA (Merck, Darmstadt, Germany), and 1 mg/mL Glucose (Sigma-Aldrich). Cells were seeded on 75 cm^2^ flasks (Sarstedt) with a 1:4 ratio or seeded into 24-well plates with 100,000 cells per mL (cells were counted using a trypan-blue exclusion assay).

Primary porcine microglia were prepared as described by Klettner et al., 2014, with modifications as described by Zhang et al., 2021 [37,59]. In brief, after removing the cornea, lens, iris and vitreous body, retinae were detached with tweezers and placed into PBS. The tissue was incubated with 5 mg/mL collagenase (Pan-Biotech), 5 mg/mL hyaluronidase (Sigma-Aldrich), and 1 mg/mL DNase (AppliChem, Darmstadt, Germany) for 40 min. Cells were washed and seeded onto 75 cm^2^ flasks, then coated with 100 µg/mL Poly-D-Lysin (Sigma-Aldrich). The media consisted of Gibco DMEM, 10% heat-inactivated fetal bovine serum, and 1% penicillin/streptomycin. Primary microglia were incubated for three to four weeks. By tapping the flask vigorously on a hard surface, cells were detached and could be seeded into 12-well plates with density of 250,000 cells/mL. For Coomassie staining, 150,000 cells/mL, and for RNA isolation, 750,000 cells/mL were seeded.

For Iba-1 staining, Coomassie staining, and the phagocytosis assay, primary microglia cells were seeded on cover slips (Th. Geyer, Renningen, Germany) in 12-well plates, which were coated with collagen I (designated as collagen A by Pan-Biotech) according to the manufacturer’s instructions.

Cell cultures were incubated at 37 °C and 5% CO_2_ in a humidified incubator. The medium was changed twice a week.

### 4.3. Stimulation

Primary microglia and SIM-A9 were seeded into 12-well or 24-well plates, respectively, and incubated for 24 h. Cells were treated directly with 50 µg/mL fucoidans and/or 1 µg/mL LPS from *E. coli* O55:B5 (Sigma-Aldrich) 30 min later than fucoidans for 24 h (SIM-A9 also 72 h).

Regarding RPE-related experiments, RPE cells were treated with LPS and fucoidans for three days. The supernatants were analyzed in ELISA or used to directly stimulate SIM-A9 or primary microglial cells for 24 h (RPE interaction tests, “RPE^+^ supernatants”). Non-stimulated RPE supernatants were collected for three days as well (before stimulation) to be used to prepare stimulation solutions for microglia with fucoidans and LPS (no RPE interaction, “RPE^−^ supernatants”). This was necessary to eliminate the effects of the different media used for RPE and microglia and for control reasons. For a better understanding of the process, example stimulation schemes with stimulation codes for this study are displayed in Table 5 and Table 6.

### 4.4. Staining

To test for microglia identity, Iba-1 was marked [35]. Primary microglia and SIM-A9 cells were seeded on cover slips. Cells were fixated with Zamboni solution for 30 min (Morphisto, Offenbach am Main, Germany). Cells were permeabilized by incubation with methanol for 30 min. Goat antibody against Iba-1 (Abcam, Cambridge, UK) was used at a concentration of 1:500 for 60 min. After washing, Alexa Fluor 555 anti-goat IgG (Thermo Fisher Scientific) in a dilution of 1:700 together with 1:500 Bisbendizimide (Sigma-Aldrich) was applied for 60 min. Cells were washed and mounted with Fluoromount G (SouthernBiotech, Birmingham, AL, USA). Images were taken with a fluorescence microscope Axio Imager.M2 and Software Zen2 blue edition, version 3.7.97.02000 (Carl Zeiss, Oberkochen, Germany). Five pictures per slide were taken. The numbers of positive and negative cells were counted.

To determine the size and morphology of the microglia, light-imaging of the cells was performed after staining with Coomassie as described in Klettner et al. 2020 [36]. Primary microglia and SIM-A9 were seeded on collagen-coated cover slides. Cells were fixated with 5% glutardialdeyhde for 40 min (Merck) and stained with Coomassie Brilliant Blue R-250 (Bio-Rad Laboratories, Hercules, CA, USA) for 40 min. Coomassie Brilliant Blue R-250 after which Destaining Solution was applied for 40 min. Cells were washed with PBS and aqua bidest (Fresenius, Bad Homburg vor der Höhe, Germany) and put into Fluoromount-G mounting medium. Three to five pictures per slide were taken using an Axiovert 100 microscope (Carl-Zeiss, Oberkochen, Germany). The number and size of the cells were determined with ImageJ2, version 1.54i, 03 March 2024 (Wayne Rashband, National Institute of Health, Bethesda, MD, USA).

### 4.5. Cell Viability

For assessing cell viability, the MTT assay was performed as described previously [36,60]. Cells were treated with 0.5 mg/mL MTT solution for 30 min, centrifugated, resuspended in DMSO (Carl Roth, Karlsruhe, Germany), and shaken for ten minutes. Measurement was performed with a microplate reader ELx800 at 550 nm and Software Gen5, version 1.11.5 (both BioTek Instruments, Winooski, VT, USA). MTT data were also used to normalize the cytokine secretion data of the ELISA.

### 4.6. Enzyme-Linked Immunosorbent Assay

Supernatants of microglia cells were collected after 24 h and for RPE after three days. For detecting mouse TNFα as well as porcine IL8, IL1β, and TNFα, corresponding DuoSet and Quantikine ELISA Kits were used according to the instructions of the manufacturer (all R&D Systems, Minneapolis, MN, USA). Measurements were taken with a microplate reader ELx800 at 450 nm. If RPE supernatants were used to stimulate microglia cells, secreted cytokines in RPE supernatants were subtracted from the microglia-secreted cytokines to normalize the microglia secretion.

### 4.7. Real-Time Polymerase Chain Reaction

RNA from SIM-A9 and primary microglia were isolated with a NucleoSpin RNA Mini Kit (Macherey-Nagel, Düren, Germany) according to the manufacturer‘s instructions. RNA was solved in 20 µL RNase-free water. The purity and concentration of RNA were assessed with NanoDrop One (Thermo Fisher Scientific). cDNA was generated with a High-Capacity cDNA Reverse Transcription Kit (Thermo Fisher Scientific) according to the manufacturer‘s instructions. Real-time PCR was performed by using TaqMan Fast Advanced Master Mix and gene expression assays [dye label 5(6)-carboxyfluorescein-minor groove binder (FAM-MGB)] according to the instructions of Thermo Fisher Scientific. *NOS2* (SS03374608_u1) and *CXCL8* (SS03392437_m1) were used as primers for primary microglial samples. *GAPDH* (SS03375629) was used as an endogenous control. *NOS2* (Mm00440502_m1) and *GAPDH* (Mm99999915_g1) was used for SIM-A9 samples. Triplicates were applied on MicroAmp Fast 96-Well Reaction plates (Thermo Fisher Scientific) and measured with QuantStudio 3 and QuantStudio™ Design & Analysis Software, version 1.4.3 (both Thermo Fisher Scientific). Thermo Fisher Connect with an RQ module was used for evaluation. The ΔΔCT method [61] was used, which calculates relative normalized gene expression with ΔCT (=CT [gene of interest] − CT [housekeeping gene]), ΔΔCT (=ΔCT [treated sample] − ΔCT [untreated sample]), and relative fold gene expression level RQ (=2^−ΔΔCT^).

### 4.8. Phagocytosis Assay

Phagocytosis assays was performed as previously described [37]. Primary microglia were treated with 2.5 µL latex beads per 1000 µL medium (Sigma-Aldrich; 1 µm diameter) for two hours at 37 °C. Cells were washed with 1% PBS-Azide (Honeywell Specialty Chemicals Seelze, Seelze, Germany) for two minutes, followed by washing and fixation with 5% paraformaldehyde (Carl-Roth) for ten minutes. Cells were cooled with 1:1 ethanol/aceton solution (Carl Roth and Merck) in the freezer at −20 °C. After washing twice with 0.2% glycerine and 0.1% albumin (Serva Electrophoresis, Heidelberg, Germany) in tris-buffered saline, cells were incubated overnight in Fluoromount G. Five pictures per well were taken using Imager.M2 and Zen 2 blue edition. For detecting phagocyted beads and cell nuclei, measurements were taken at 525 nm and 465 nm, respectively.

### 4.9. Statistical Analysis

To summarize the data and to generate figures, Microsoft Excel and PowerPoint were used (Microsoft Office 2010, Microsoft, Redmond, WA, USA). GraphPad Prism 9 (GraphPad Software, Inc., San Diego, CA, USA) was used to determine normality and *p*-values between groups. In general, the significance calculation was considered as two-tailed and non-matching. Normality was checked using the Shapiro–Wilk test. For parametric data, the one-sample *t*-test was used to calculate the significance against fixed values, and ANOVA (analysis of variance) with post hoc multiple comparison tests (Dunnett’s test) was performed to determine *p*-values between groups. For comparisons between two groups, Student’s *t*-test was used. For non-parametric data, the Kruskal–Wallis test followed by multiple comparison tests (Dunn’s test) was conducted. For parametric data, the paired Student’s *t*-test was performed to determine the significance between untreated RPE^−^- and RPE^+^- treated supernatant stimulation for the same condition/well. PCR data statistics and *p*-values (student’s *t*-test) were calculated via Thermo Fisher Connect. All data were determined as parametric data and evaluated as indicated above, besides the data for phagocytosis and morphology, which were considered as non-parametric. Data were considered as significant to the compared groups if *p* was <0.05.

## 5. Conclusions

The aim of this study was to test the possible anti-inflammatory effects of fucoidans and fucoidan-treated retinal pigment epithelium on microglia cells. The proinflammatory properties of activated primary microglia and the induction by LPS were clearly demonstrated. Phagocytosis was reduced under treatment with FuBB04. Fucoidans displayed limited anti-inflammatory activities in the microglia models utilized, initially even stimulating the production and expression of certain inflammatory mediators or reducing the viability depending on the microglia model and stimulation time. In primary microglia, however, the fucoidans reduced the activation of primary microglia, as indicated by the cell size. RPE reduced the proinflammatory activation of SIM-A9 and primary retinal microglia with or without fucoidan treatment. Fucoidans alone showed no significant anti-inflammatory effects on microglia. In addition, we were able to confirm the anti-inflammatory effect of RPE on microglia, which was also shown for fucoidan-treated RPE.

## Figures and Tables

**Table 1:** Effects of fucoidans and LPS on *NOS2* gene expression in SIM-A9. Mouse microglial cell line SIM-A9 was exposed to 50 µg/mL fucoidans FVs, Fuc1, and FucBB04 30 min prior to treatment with 1 µg/mL lipopolysaccharide (LPS) for 24 h (**left**) or 72 h (**right**). qPCR was performed to measure the gene expression of nitric oxide synthase 2 (*NOS2*). Expression was normalized by measuring *GAPDH* expression. Relative quantification factor Rq of biological replicates with minimum and maximum values is pictured relative to no fucoidan control (“Control”) and LPS, set to 1.000. Student’s *t*-test was used to determine significances between groups. *n* = 6. *p*-values were calculated against “Control” and “LPS”. * *p* < 0.05, ** *p* < 0.01 compared to “Control”.

24 h					72 h				
Bio Group	Rq	Rq Min	Rq Max	*p*-Value	Bio Group	Rq	Rq Min	Rq Max	*p*-Value
Control	1.000	0.335	2.988	1.000	Control	1.000	0.652	1.533	1.000
FVs	3.189	1.794	5.671	0.146	FVs	0.809	0.324	2.022	0.703
Fuc1	3.128	1.658	5.901	0.155	Fuc1	3.871	1.307	11.470	0.084
FucBB04	4.292	3.066	6.009	0.071	FucBB04	0.121	0.008	1.853	0.250
LPS	7.422	4.803	11.466	0.035 *	LPS	6.015	3.331	10.862	0.006 **
FVs + LPS	3.922	2.065	7.445	0.102	FVs + LPS	4.013	2.580	6.243	0.017 *
Fuc1 + LPS	3.904	1.822	8.364	0.109	Fuc1 + LPS	8.056	5.069	12.802	0.002 **
FucBB04 + LPS	9.229	4.589	18.843	0.018 *	FucBB04 + LPS	7.489	4.792	11.704	0.106
LPS	1.000	0.718	1.408	1.000	LPS	1.000	0.562	1.781	1.000
FVs + LPS	0.528	0.278	1.003	0.178	FVs + LPS	0.667	0.429	1.038	0.348
Fuc1 + LPS	0.526	0.245	1.127	0.220	Fuc1 + LPS	1.339	0.843	2.128	0.468
FucBB04 + LPS	1.351	0.667	2.738	0.468	FucBB04 + LPS	0.687	0.440	1.074	0.398

**Table 2:** Effects of fucoidans and LPS on *NOS2* and *CXCL8* gene expression in primary microglia. Primary porcine microglia were exposed to 50 µg/mL fucoidans FVs and FucBB04 30 min prior to treatment with 1 µg/mL lipopolysaccharide (LPS) for 24 h. qPCR was performed to measure gene expression of interleukin 8 (*CXCL8*, **left**) and nitric oxide synthase 2 (*NOS2*, **right**). Expression was normalized by measuring *GAPDH* expression. Relative quantification factor Rq of biological replicates with minimum and maximum values are depictured relatively to no fucoidan control (“Control”) and LPS, set to 1.000. Student’s *t*-test from Thermo Fisher Connect was used to determine significances between groups. *n* = 6. *p*-values were calculated against “Control” and LPS. ** *p* < 0.01 compared to “Control”.

*CXCL8*					*NOS2*				
Bio Group	Rq	Rq Min	Rq Max	p-Value	Bio Group	Rq	Rq Min	Rq Max	p-Value
Control	1.000	0.050	20.002	1.000	Control	1.000	0.121	8.292	1.000
FVs	0.250	0.034	1.772	0.351	FVs	0.740	0.371	1.464	0.732
FucBB04	7.640	1.588	36.739	0.160	FucBB04	1.450	0.297	7.021	0.737
LPS	109.550	18.181	660.034	0.005 **	LPS	20.460	6.705	62.405	0.009 **
FVs + LPS	137.650	31.404	603.344	0.004 **	FVs + LPS	28.850	16.398	50.762	0.005 **
FucBB04 + LPS	659.110	258.358	1681.481	0.001 **	FucBB04 + LPS	10.738	1.949	59.160	0.079
LPS	1.000	0.166	6.025	1.000	LPS	1.000	0.328	3.051	1.000
FVs + LPS	1.257	0.287	5.508	0.815	FVs + LPS	1.410	0.802	2.482	0.501
FucBB04 + LPS	6.017	2.358	15.350	0.076	FucBB04 + LPS	0.525	0.095	2.892	0.531

**Table 3:** Effects of RPE supernatants on *NOS2* gene expression in SIM-A9. Mouse microglia cell line SIM-A9 was exposed to 50 µg/mL FVs and FucBB04 30 min prior to treatment with 1 µg/mL lipopolysaccharide (LPS) for 24 h (RPE^−^), or by using instead RPE supernatants (RPE^+^) stimulated (by the same agents) for three days. qPCR was performed to measure the gene expression of nitric oxide synthase 2 (*NOS2*). Expression was normalized by measuring *GAPDH* expression. Relative quantification factors Rq of biological replicates with minimum and maximum values are pictured relative to no fucoidan control (“Control”) or LPS, set to 1.000. Student’s *t*-test from Thermo Fisher Connect was used to determine significances between groups. *n* = 6. *p*-values were calculated against “Control” and “LPS” (RPE^−^) or “Control” and “LPS” control from treated RPE (RPE^+^). * *p* < 0.05, ** *p* < 0.01, *** *p* < 0.001 compared to “Control” (RPE^−^) or “Control” (RPE^+^).

Bio Group	Rq	Rq Min	Rq Max	*p*-Value (RPE^−^)	*p*-Value (RPE^+^)
Control^−^	1.000	0.668	1.496	1.000	0.619
FVs^−^	2.371	1.512	3.718	0.047 *	0.037 *
FucBB04^−^	5.011	2.322	10.814	0.048 *	0.035 *
LPS^−^	41.913	30.695	57.231	<0.001 ***	0.001 **
FVs + LPS^−^	30.788	22.812	41.554	<0.001 ***	0.001 **
FucBB04 + LPS^−^	22.226	12.579	39.270	0.002 **	0.002 **
Control^+^	0.824	0.513	1.324	0.619	1.000
FVs^+^	1.318	0.856	2.027	0.542	0.351
FucBB04^+^	2.237	0.663	7.548	0.372	0.290
LPS^+^	17.843	10.538	30.213	0.002 **	0.002 **
FVs + LPS^+^	43.195	35.178	53.039	0.001 **	0.001 **
FucBB04 + LPS^+^	23.564	18.223	30.471	0.001 **	0.002 **
LPS^−^	1.000	0.732	1.365	1.000	0.088
FVs + LPS^−^	0.735	0.544	0.991	0.253	0.207
FucBB04 + LPS^−^	0.530	0.300	0.937	0.186	0.650
LPS^+^	0.426	0.251	0.721	0.088	1.000
FVs + LPS^+^	1.031	0.839	1.265	0.905	0.087
FucBB04 + LPS^+^	0.562	0.435	0.727	0.071	0.473

**Table 4:** Algal species, molecular weight, fucose content, molar degree of sulfation, and provider.

Fucoidan	Algae Species	Molecular Weight	Fucose Content	Degree of Sulfation	Provider
FVs	*Fucus vesiculosus*	50 kDa	88 mol%	0.6	Sigma-Aldrich
Fuc1	*Laminaria hyperborea*	1548 kDa	97 mol%	1.7	Alginor ASA
FucBB04	*Laminaria hyperborea*	3700 kDa	86 mol%	0.9	Alginor ASA

**Table 5:** Stimulation scheme for RPE plates related to the RPE–microglia interaction experiments. Depicted is an example scheme for a 12-well plate seeded with primary porcine retinal pigment epithelium cells (RPE). After two weeks of incubating and reaching confluence, the medium was exchanged and supernatants were collected after three days (“RPE^−^ supernatants”). After three weeks, the same wells were stimulated with 50 µg/mL fucoidans FVs, Fuc1, or FucBB04 and/or 1 µg/mL lipopolysaccharide (LPS) for three days, and supernatants were collected again (“RPE^+^ supernatants”). Stimulated RPE^+^ supernatants were used to stimulate microglia for 24 h. RPE^−^ supernatants were used to prepare stimulation solutions with LPS and fucoidans to stimulate the microglia without RPE pre-stimulation, for comparison reasons.

12-well plate	1	2	3	4
A	Control	FVs	Fuc1	FucBB04
B	LPS	FVs + LPS	Fuc1 + LPS	FucBB04 + LPS
C				

**Table 6:** Stimulation scheme for the microglia plated with RPE supernatants regarding RPE–microglia interaction experiments. Depicted is an example scheme for the retinal pigment epithelium (RPE) interaction experiments with microglia. RPE supernatants were prepared as described in Table 5. Microglia were seeded into 24-well plates for 24 h and stimulated with RPE supernatants. These RPE supernatants were collected from RPE-stimulated wells (with fucoidans and/or LPS) marked with a “+” and used for direct stimulation of the microglia. Before RPE stimulation, supernatants were collected from the same wells for three days but without stimulation, then marked with a “−”. The RPE^−^ supernatants were used to prepare stimulation solutions with LPS and fucoidans. A control with just microglia medium was used to check the compatibility of the RPE media used for stimulating the microglia.

	RPE^−^ treatment			RPE^+^ treatment		
24-well plate	1	2	3	4	5	6
A		FVs (RPE^−^)	FVs + LPS (RPE^−^)		FVs (RPE^+^)	FVs + LPS (RPE^+^)
B	Control (RPE^−^)	Fuc1 (RPE^−^)	Fuc1 + LPS (RPE^−^)	Control (RPE^+^)	Fuc1 (RPE^+^)	Fuc1 + LPS (RPE^+^)
C	LPS (RPE^−^)	FucBB04 (RPE^−^)	FucBB04+LPS (RPE^−^)	LPS (RPE^+^)	FucBB04 (RPE^+^)	FucBB04 + LPS (RPE^+^)
D	Microglia medium control					

## Data Availability

Data are available upon request.
